# Editorial: Activation of the cGAS-STING pathway by extra-nuclear DNA and its pharmacognostic modulation in human disease

**DOI:** 10.3389/fphar.2025.1519429

**Published:** 2025-01-29

**Authors:** Preston M. McCourt, Charles A. Day, Souren Paul

**Affiliations:** ^1^ The Hormel Institute, University of Minnesota, Austin, MN, United States; ^2^ Cell and Molecular Biology Lab, TERI School of Advanced Studies, Vasant Kunj, New Delhi, India

**Keywords:** cGAS, STING, cancer, IRF3, IFN-β, IFN1, gastrointestinal (GI) diseases, radiation therapy

Centromere defects, chromosome instability, and concomitant cGAS-STING pathway activation correlate with increased fibrosis markers, suggesting that the cGAS-STING pathway is linked to immune modulation in human diseases (Paul et al., 2022; Contreras-Galindo et al., 2023). This Research Topic fostered a multidisciplinary understanding of cGAS-STING pathway activation in human diseases. Additionally, it aimed to highlight advancements in cGAS-STING modulators, contributing to drug discovery efforts for the treatment of autoimmune diseases and cancer.

The detection of extranuclear DNA, whether self or foreign, by Cyclic GMP-AMP synthase (cGAS) plays a vital role in human health (Dvorkin et al., 2024). When cGAS binds to extranuclear DNA, it stimulates the production of the second messenger cyclic guanosine monophosphate (cGMP), which activates the stimulator of interferon genes (STING). STING activation triggers various cellular responses, including the activation of interferon regulatory factor 3 (IRF3) and the release of interferons (Hopfner and Hornung, 2020). cGAS-STING pathway activation can lead to several outcomes, such as cell cycle arrest, apoptosis, and the recruitment of the immune system (Decout et al., 2021). Recent findings suggest that chromosome segregation defects can activate the cGAS-STING pathway in systemic sclerosis, potentially contributing to abnormal autoimmune responses (Paul et al., 2022). Significant efforts are underway to discover specific and effective cGAS-STING inhibitors as researchers aim to blunt the cGAS-STING pathway in auto-immune disorders. A recent study showed that flavonoids are effective against the cGAS-STING pathway (Li et al., 2023) and, in addition, flavonoids are known to have strong anti-inflammatory activities (Gonfa et al., 2023). This research Research Topic also highlights efficacy of Licorice extract and polysaccharide from *Glycyrrhiza uralensis* against cGAS-STING pathway.

Conversely, cGAS-STING agonists may offer therapeutic benefits; a recent study demonstrated that activating this pathway induces IFN-β and primes CD8^+^ T cells to target tumor cells, highlighting its potential in cancer immunotherapy (Gan et al., 2022). However, some research indicates that the cGAS-STING pathway may also facilitate tumorigenesis and metastasis (Kwon and Bakhoum, 2020).

Chen et al., offered a comprehensive review on the role of cGAS-STING in liver diseases such as viral hepatits B and hepatocellular carcinoma. During viral hepatitis, cGAS-STING signaling, and cytokine synthesis play a pivotal role in the antiviral-activity of hepatocytes. In hepatocellular carcinoma, the cGAS-STING pathway is activated by DNA damage, leading to IFN-1 release which in turn activates tumor-specific CD8^+^ T cell and helps induce systemic tumor immunity. Moreover, the authors suggest that the activation of the cGAS-IRF3 pathway is positively correlated with the severity of alcoholic liver disease (ALD) and non-alcoholic fatty liver disease (NAFLD).

Another comprehensive review article by Ramos et al. discusses the role of cGAS in various gastrointestinal (GI) diseases including inflammatory bowel disease (IBD) and in GI malignancy. The authors explain the contribution of cGAS-mediated autophagy activation on intestinal epithelial cell integrity and innate immunity responses. Interestingly, cGAS shows a contradictory role in GI-related cancers, showing both oncogenic and anti-oncogenic functions. Ramos et al. also discuss non-canonical activation of cGAS in various GI-related diseases. STING-independent activation of cGAS includes an interaction between cGAS and Beclin-1, autophagy activation and degradation of pathogenic DNA. On another note, cGAS mediated autophagy of micronuclei shows an interaction between cGAS and essential autophagy protein LC3. DNA damage in the nucleus can also trigger nuclear translocation of cGAS which inhibits homologous recombination of double strand breaks by interacting with PARP-1. The authors also summarize the potential antagonists and agonists used against cGAS in various cell lines and mouse models.

In another cGAS-STING review, Colangelo et al. explain the importance of cGAS-STING signaling in radiation therapy. The review gives an overview of the cGAS-STING pathway in tumor microenvironment after ionizing radiation exposure. DNA damage (nuclear or mitochondrial) from ionizing radiation releases to cytosol within a short period of time and activates cGAS-STING. The authors discuss the importance of immune cells in terms of STING activation, whether activated directly or by tumor-derived cGAMP. STING activation further activates downstream IRF3 signaling and eventually IFN-1, which mediates important immune signaling response in the tumor microenvironment. IFN-1 has been shown to facilitate anti-tumor immunity through interferon α/β receptor 1 (IFNAR1) on CD8α^+^ dendritic cells. The authors explain the importance of dendritic cells in a tumor microenvironment in terms of cGAS-STING activation. cGAMP synthesized by tumor cells sometimes neutralizes by ENPP1 present in tumor microenvironment or can be taken up by DC to activate STING (Wang et al., 2023). With these observations, the authors support the hypothesis that dendritic cells are immensely important in promoting antitumor immunity in response to radiation in a tumor tissue microenvironment. Colangelo et al. explore the encouraging outcomes of combining radiation therapy with STING agonists in preclinical studies and discuss important factors that could shape the design of future clinical trials. They mention that larger doses of radiation are less effective than multiple fractions of lower doses in activating cGAS. Interestingly, larger doses of radiation can induce cytosolic TREX1, which can neutralize released DNA and prevent cGAS activation. In the later part of the review authors included the preclinical results of radiation therapy with STING agonists.

A research article from this Research Topic, published by Luo et al., demonstrates the effectiveness of licorice extract against cGAS-STING pathway activation. Licorice, derived from *G. uralensis* Fisch., is a Chinese herbal medicine known for its anti-inflammatory, immunomodulatory, anti-cancer, and hepatoprotective effects (Foghis et al., 2023). Non-alcoholic steatohepatitis (NASH) is a severe and progressive form of non-alcoholic liver disease (Kabarra et al., 2021). As the authors mention in this manuscript, the cGAS-STING pathway is highly important in the progression of NASH. The authors treated bone marrow-derived macrophages (BMDM) and THP-1 cells with various concentrations of licorice extract and then conducted ELISA assays for IFN-β and Western blotting for IRF3, pIRF3, and STING. They also analyzed the active compounds in the licorice extract, finding that it primarily comprises flavonoids. The licorice extract exhibits inhibitory effects on the DNA-triggered STING signaling pathway in a dose-dependent manner. Using immunofluorescence, the authors conclude that licorice extract significantly reduces IRF3 nuclear translocation induced by cGAMP. They further discovered that licorice extract inhibits STING oligomerization. Additionally, using an animal model, the authors confirm the inhibition of hepatocyte inflammation and fibrosis associated with NASH.

Interestingly, another research article on *G. uralensis* Fisch. shows a significant inhibitory effect of its metabolites on cGAS-STING pathway activation. Hui et al. discovered that *G. uralensis* polysaccharide (GUP) exhibits a strong inhibitory effect on cGAS-STING signaling, as analyzed by Western blotting and polymerase chain reactions. Unlike the previous paper by Luo et al., they also used bone marrow-derived macrophages (BMDM) and THP-1 cells, and they additionally confirmed their hypothesis in peripheral blood mononuclear cells (PBMC). The authors analyzed a similar mechanism as in the previous study and found that GUP has no effect on STING oligomerization. Rather, through immunoprecipitation experiments, they confirmed that GUP interferes with STING-TBK1 and TBK1-IRF3 interactions. By using STING agonist DMXAA-induced and CLP-induced sepsis animal models, they confirmed the effectiveness of GUP on cGAS-STING signaling.
